# The transfer and implementation of an Aboriginal Australian wellbeing program: a grounded theory study

**DOI:** 10.1186/1748-5908-8-129

**Published:** 2013-10-31

**Authors:** Janya R McCalman

**Affiliations:** 1 The Cairns Institute, James Cook University, PO Box 6811, Cairns 4870, Australia

**Keywords:** Indigenous, Aboriginal, Program, Transfer, Spread, Implementation, Grounded theory

## Abstract

**Background:**

The concepts and standard practices of implementation, largely originating in developed countries, cannot necessarily be simply transferred into diverse cultural contexts. There has been relative inattention in the implementation science literature paid to the implementation of interventions targeting minority Indigenous populations within developed countries. This suggests that the implementation literature may be bypassing population groups within developed countries who suffer some of the greatest disadvantage. Within the context of Aboriginal Australian health improvement, this study considers the impact of political and cultural issues by examining the transfer and implementation of the Family Wellbeing program across 56 places over a 20-year period.

**Methods:**

A theoretical model of program transfer was developed using constructivist-grounded theory methods. Data were generated by conducting in-depth interviews with 18 Aboriginal and non-Aboriginal research respondents who had been active in transferring the program. Data were categorised into higher order abstract concepts and the core impetus for and process of program transfer were identified.

**Results:**

Organizations transferred the program by using it as a vehicle for supporting inside-out empowerment. The impetus to support inside-out empowerment referred to support for Aboriginal people's participation, responsibility for and control of their own affairs, and the associated ripple effects to family members, organizations, communities, and ultimately reconciliation with Australian society at large. Program transfer occurred through a multi-levelled process of embracing relatedness which included relatedness with self, others, and structural conditions; all three were necessary at both individual and organizational levels.

**Conclusions:**

Similar to international implementation models, the model of supporting inside-out empowerment by embracing relatedness involved individuals, organizations, and interpersonal and inter-organizational networks. However, the model suggests that for minority Indigenous populations within developed countries, implementation approaches may require greater attention to the empowering nature of the intervention and its implementation, and multiple levels of relatedness by individuals and organizations with self, others, and the structural conditions. Key elements of the theoretical model provide a useful blueprint to inform the transfer of other empowerment programs to minority Indigenous and other disadvantaged populations on a case-by-case basis.

## Background

There has been an accelerated growth in implementation science literature in western countries in recent decades based on the insight that research findings should be more widely implemented in health practice [1]. Researchers have developed field-specific theories, models, and frameworks, many of which are specifically intended to help in planning implementation processes. Examples of such theories are the Promoting Action on Research Implementation in Health Services model [2], the Consolidated Framework for Implementation Research [3], and the Conceptual Model for Considering the Determinants of Diffusion, Dissemination and Implementation of Innovations in Health Service Delivery and Organization [4]. A consistent theme across these theories, models, and frameworks is the importance of ensuring a good 'fit’ between the process of implementation, the innovation proposed for adoption, and the context (including the structural conditions, organizational settings, and individuals involved). However, although these models may provide a useful starting point, the concepts and standard practices of implementation, largely originating in developed countries, cannot necessarily be directly translated into diverse cultural contexts or to disadvantaged populations. There has been relative inattention in the implementation science literature paid to the implementation of interventions targeting minority Indigenous populations within developed countries.

The significant gaps in health and wellbeing equity between Indigenous and non-Indigenous populations in Canada, North America, New Zealand, and Australia have been well described; as has been evidence of achievement of health targets that has shown that it is possible to improve health [5]. However, a paucity of intervention and implementation literature in Indigenous health means that we have not identified and tested effective strategies for change [6]. Nor have we researched processes for their effective implementation [7,8]. For example, a search of this journal using the search terms Indigenous or Maori or native American or first nations or Aborigin* or Torres or Metis, found only six papers relating to the implementation of innovations for these Indigenous populations [9-14]. This suggests that the implementation literature may be bypassing population groups within developed countries who suffer some of the greatest disadvantage.

This study developed a grounded theoretical model of the transfer and implementation of the Family Wellbeing Program (FWB) across 56 places in Australia over a 20-year period. Transfer was considered to be the process whereby FWB was made available and accessible to new settings through the interactive engagement for change co-produced by program providers, implementing organizations and funders; while implementation was the process of program uptake and routinization within a new organization [4,15,16]. In the case of FWB, transfer had occurred through an episode-by-episode response to demand; rather than through top-down dissemination by governments. Greenhalgh *et al.*[17] cited a clear need for such empirical studies that explore the processes by which innovations are implemented and sustained (or not) in particular contexts and across health service organizations, and how such processes can be enhanced.

## Methods

### Study design

The study was conducted within the overarching framework of the values-based Empowerment Research Program (ERP) at James Cook University in Cairns. The theorist was a non-Aboriginal researcher who had worked in the ERP for six years, and who had knowledge of episodes of FWB transfer as well as established research relationships with many of those engaged in program transfer and implementation. The ERP’s strengths-based research approach was applied within this study to enhance the efforts of those involved in transferring and implementing the FWB program [18]. Such strengths-based approaches adhere to the principles of decolonizing research methodologies, which have been developed by global Indigenous scholars over the past two decades as a way of reclaiming the validity of Indigenous ontologies and epistemologies in research endeavors [19,20].

Constructivist-grounded theory methods were used to theorize the processes underlying FWB transfer [21]. Constructivist-grounded theory is considered appropriate to the task of conducting exploratory research in situations, such as Aboriginal program transfer and implementation, where there has been little prior research because it is generally based on interviews with those directly affected by the phenomenon and derives a theory grounded in their perspectives and experiences. The methods are also well suited to encompassing the particular ethics of care and responsibility that are requisite in Aboriginal research methodologies [22]. The protocol for the research project was approved by JCU Ethics Committee (H3532).

### The intervention

FWB is a nationally-accredited training program through the Australian vocational education and training sector. It provides Aboriginal Australian students with pathways to employment and further training in youth work, community services, health, and education. Skills taught included foundational counselling skills for coping with personal and community problems including grief and loss. However, the complex nature of Aboriginal health and wellbeing issues and their determinants called for more than just a standard didactic training program.

FWB was designed to provide an empowering framework within which participants were supported to interact, identify personal, professional, and community wellbeing priorities, and tackle a variety of issues. The impact of colonization on people's lives is acknowledged and participants are asked questions such as: 'How can we heal our wounds? Who are we? Why we are here and what are our beliefs? What to do and how to do it?’ [23]. Such questions elicit program participants’ reflections on their physical, emotional, mental, and spiritual health needs; relationship patterns; and experiences throughout their life journey as well as their qualities and strengths that promote resilience, the identification of goals for personal change, and consideration of agency for change in their families and communities. Hence, FWB provides a forum within which Aboriginal participants gain understanding and control of their lives as a necessary first step to acting effectively on their decisions towards health, wellbeing and social change.

FWB has been adapted for various settings, issues, and target groups, and has been variously delivered as a community development and employment, training and capacity development, health promotion, empowerment research, and school education program. Micro-level evaluations of FWB since 2000 documented promising personal empowerment outcomes such as the development of intellectual curiosity; reflective skills; hope and confidence; an enhanced respect for self and others; improved relationships; a strengthened capacity to deal with life challenges such as substance abuse and violence; and increased engagement in broader change processes, employment and education *e.g.*[24,25]. At community and structural levels, participant groups advocated housing, childcare, education and health improvements, and FWB principles had been incorporated into state school curricula and primary healthcare services [24,26-28].

### Organizational settings

**Figure 1:**
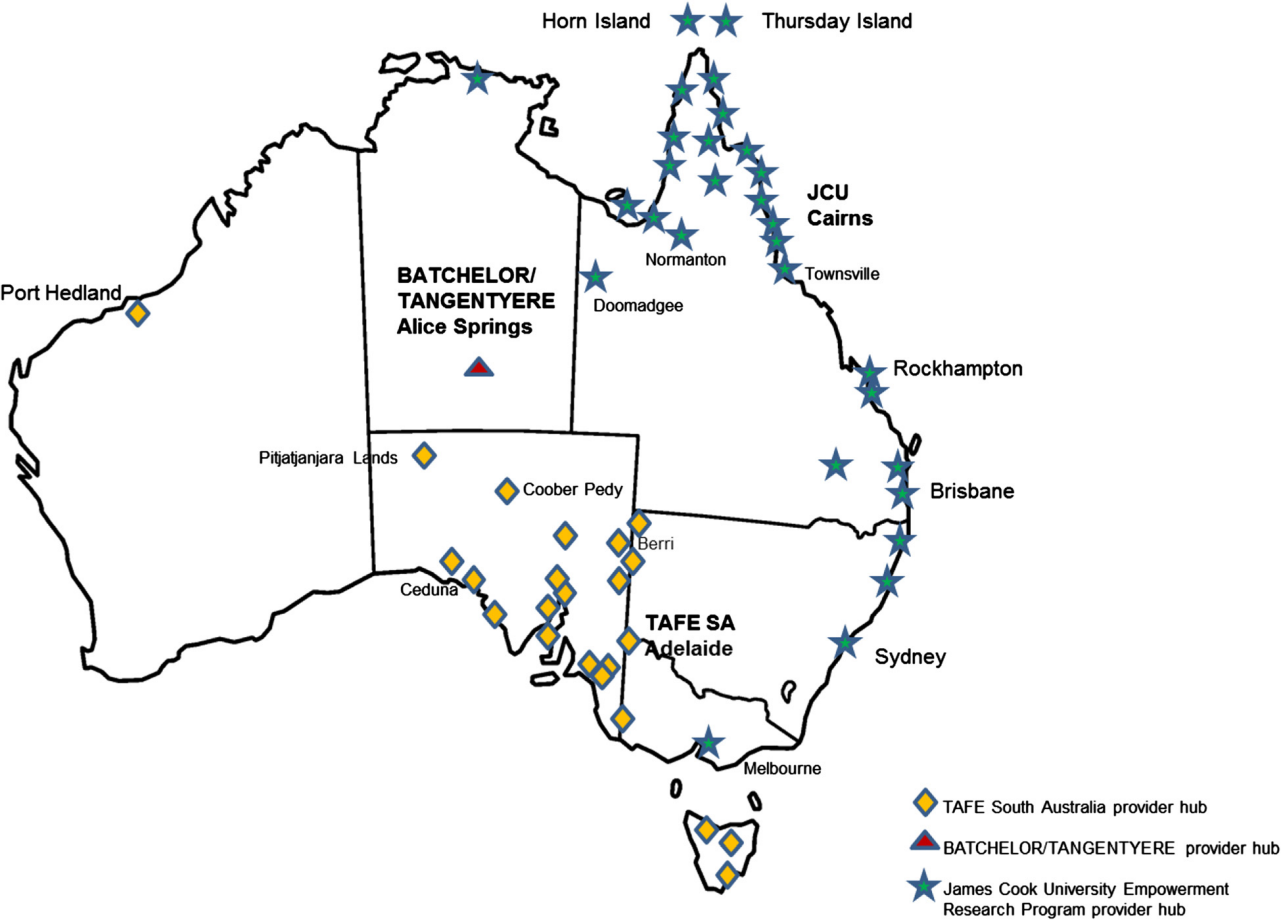
The geographical places in Australia within which FWB has been implemented.

FWB has been delivered by three main training provider hubs^a^ located in South Australia, the Northern Territory, and Queensland to primary healthcare, education, and welfare organizations in at least 56 places across Australia (Figure 1). These provider hubs and implementing organizations were all cross-cultural workplaces, encompassing dynamics of culture and power within intercultural organizational and inter-organizational encounters [29,30]. From 1992 to 2011, there were at least 206 discrete program deliveries to 3,300 participants, and 91% of FWB participants were Aboriginal [31]. The extent of FWB spread across all of Australia’s states and territories, except the Australian Capital Territory, and through non-government, government, private, and academic implementing organizations provided an opportunity to study the program transfer process across diverse geographical and organizational settings.

### The structural conditions

Australian social structures have long been associated with health inequity and widespread Aboriginal policy failure. For at least the twenty-year period since FWB was developed in 1992:

'… Australian governments have developed and funded policies and programs to improve the socio-economic status of Indigenous people, and overcome a long history of poverty and marginalization. Progress has been made. Yet in 2009, despite the formal recognition of equality so many years ago, Indigenous people remain among the most disadvantaged Australians. Many simply do not have the opportunities afforded their fellow Australians and many are not able to participate fully in our national life’ [32].

Many Australian indicators show that the past 20 years have not produced improved health, education (literacy and numeracy), child protection, adult imprisonment, or housing (overcrowding) conditions for Australia’s 500,000 Aboriginal people (2.3% of population) [33]. As a broad indicator of health, the current gap between Indigenous and non-Indigenous Australians’ life expectancy at birth is still estimated to be 11.5 years for males and 9.7 years for females [33].

Such structural conditions were not simply a passive backdrop to FWB transfer and implementation. Interactions and negotiations to transfer FWB were affected by shifting Commonwealth and state and territory Aboriginal policies and frameworks for Aboriginal development. These policies both created a need for programs such as FWB as culturally appropriate responses for tackling Aboriginal community development, employment, training, health and wellbeing, research and educational needs, and also influenced the availability of resources and support for programs such as FWB. Structural conditions from the Aboriginal domain were also influential. For example, leadership by Aboriginal Elders and others was critical for bridging the cultural interface between government bureaucratic structures and resources and Aboriginal community members to originate, adapt, and transfer FWB. Spiritual and cultural beliefs, values, and practices underpinned efforts to transfer and adapt the program.

### Research respondents

Initial sampling of research respondents was drawn from a purposive sample of five Aboriginal and non-Aboriginal people who had been active in FWB transfer and implementation across diverse organizations. Further sampling was determined by identifying those respondents who were likely to provide divergent views about the emerging theoretical issues [21,34]. In total, 18 research respondents were interviewed; they had experienced 177 of the 206 (86%) identified situations of FWB transfer, spanning from the first program delivery in 1993 to the time of interview in 2011. They included Aboriginal and non-Aboriginal researchers, facilitators, coordinators, advocates, and program developers. They represented both genders and different age groups (30 s to 60 s); important because FWB had been transferred to gender-specific men’s and women’s groups and to age-specific youth groups. All but three research respondents had also experienced FWB as program participants. At the time of the interviews, 14 of the 18 research respondents were currently engaged in FWB transfer.

### Data collection

Data were generated from in-depth interviews, varying in length from 45 minutes to two hours and 20 minutes, and conducted by the same researcher. An interview guide was adapted to explore emerging issues as theorizing progressed [21].

### Data analysis

Interviews were transcribed verbatim with transcripts imported into NVIVO and coded to identify recurrent themes and theoretical constructs [35]. The identified concepts were then categorized into higher-order abstract constructs and the relationships between constructs identified. The process of examining the concepts was repeated until the theorist was satisfied that higher-order constructs and their relationships could be modelled in such a way that explained the great majority of the data and that she had identified the central concern of those involved in program transfer and the basic process that facilitated that concern [35].

To ensure that the analytical interpretations made sense to those active in FWB transfer, the constructed theoretical model was presented to five individuals who had been involved in FWB transfer and implementation. Three had been research respondents; four were Aboriginal. Grounded theorists do not generally advise the need for returning the analysis to research respondents to validate findings; instead recognizing that research respondents’ beliefs and understandings are influenced by context and subject to change, and that the need for checking is subsumed by the grounded theory method of concurrent data generation and analysis [36]. However, given the cross-cultural context of the study, this checking was ethically responsible, respectful and important for interpretation. Given the paucity of the Aboriginal implementation literature, the significance of the theoretical model for practice and policy was then examined by comparing it with both established Aboriginal Australian and international implementation models.

## Results

The constructed model incorporates the impetus for program transfer and the process by which transfer occurred. The process encompassed three dimensions, each of which comprised several sub-processes. The model and verifying grounded qualitative data are presented below.

### The impetus: supporting inside-out empowerment

The central concern of those who were active in FWB program transfer (hereafter FWB agents) was to support inside-out empowerment. This impetus for program transfer referred to FWB agents’ participation in, responsibility for and control of their own affairs, and then through a ripple effect, their support for the empowerment of family members, organizations, communities, and ultimately reconciliation with Australian society at large. This was identified in the narrative of a non-Aboriginal researcher who recalled:

'Where the penny dropped for me, really was… the notion of integrating personal empowerment and community empowerment; that the two go hand in hand. Unless you can focus on asking people basic questions: Who am I? Where is my place now in relation to my broader community? Then it’s hard to just focus on either the personal or the community. So it struck me that this program was trying to do this.’

Thus, the individual and broader organizational and community manifestations of supporting inside-out empowerment were closely interwoven.

### The process: embracing relatedness

**Figure 2:**
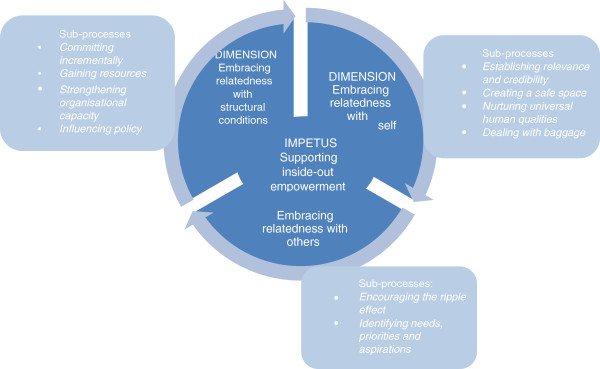
The theoretical model: Supporting inside-out empowerment by embracing relatedness.

Organizations (and their individual employees) undertook a process of embracing relatedness to transfer the program. Embracing relatedness was three-dimensional: relatedness with self (purpose, spiritual and cultural values and beliefs, leadership, principles, capacity, and control); other organizations (partnerships, networks including family and other informal networks); and the structural conditions inherent within situations of program transfer (leadership, government policies, accountabilities, and resources, particularly funding). Shifts in each of the three dimensions of relatedness were prompted within each episode of FWB transfer and implementation, and occurred through interactions across episodes of FWB transfer. Figure 2 depicts the central concern of supporting inside-out empowerment as central to relatedness with self, others and the structural conditions, and the sub-processes associated with each dimension of embracing relatedness.

The first dimension of the process of program transfer was embracing relatedness with self. For organizations, this occurred through negotiating vision and purpose, principles for practice, and capacity strengthening. A non-Aboriginal FWB program manager elucidated: 'So in the first instance it was for my team … What it was about for me was: one was to really create a much more healthier workplace culture, to build teamwork.’ Parallel with organizational processes, individual FWB program participants also embraced relatedness with self, initially by becoming engaged in the program. This engagement was enhanced by the program’s relevance and credibility, trustworthiness and the universal human qualities that it nurtured. An Aboriginal facilitator and researcher reflected:

'Our mob when they hear that it’s been developed by our own people, that’s the only reason why sometimes I think they come along to it. So I think that’s the most critical thing. And that it works of course, but you know, people don’t know that it’s going to work until they’ve done it. But to get them there is, you know, that’s just so, so important; that it is developed by Aboriginal people.’

Similarly, another Aboriginal facilitator, reflected: 'finally—we got something you can relate to without big words and having strangers from out of town.’

FWB agents’ experiential processes of having their needs met within a safe space prompted reflection and greater awareness of life goals and purpose, spirituality, cultural values and beliefs, identity, ethical practice, and agency. An Aboriginal FWB facilitator, reflected:

'It gives that two-way understanding, that’s what FWB does… We’re all at this level of understanding… it gets back to that safe space. It allows that two-way understanding to take place because it’s creating that safe place for the dialogue to occur.’

Participants’ applied the generic empowerment skills learned through the program to deal with their own 'baggage.’ An Aboriginal facilitator described her personal process of change in coming to the realization that: 'that’s what it is in the end—you know—addressing your own problems, no matter how hard they are.’ A non-Aboriginal facilitator and researcher observed that FWB participants deployed their capabilities and skills according to what was relevant at that time within 'their own challenging family and life issues.’ Another non-Aboriginal researcher observed: 'there is something generic in terms of skills or capabilities that once acquired, people acquire it, it can be applied in different settings.’ The personal experiential change processes emanating from program participation drove participants to advocate for program transfer to other settings in order to support other Aboriginal people’s inside-out empowerment.

The second dimension of the transfer process was embracing relatedness with others. Relatedness with other organizations led to the development of networks and partnerships across which to transfer the program. Relatedness with other organizations was described by a non-Aboriginal program manager:

'… to then extend it out to create partnerships with other regional organizations who were going out to communities engaging with the same people we were … that we each knew what each other was doing and complemented one another.’

In addition to organizational partnerships, individual FWB program participants were also motivated to support the empowerment of others, and used their informal networks to embrace relatedness with extended family members and others. With strengthened personal capacity, individual FWB participants were motivated to take action, supporting improvements in aspects of their family life, workplace, and community issues. A non-Aboriginal facilitator and researcher observed: 'the minute that people felt in control themselves, they were really keen to help other people.’ Applying her enhanced capability and skills to community improvement, for example, an Aboriginal researcher reflected: 'As an Aboriginal person, all I’m there for is to be able to be part of a group that will create change and lift the whole game for our people.’ Both at organizational and individual levels, needs, priorities, and aspirations were identified, and the generic FWB modules were tailored to meet those needs.

The third dimension of the transfer process was embracing relatedness with the structural conditions. For organizations, embracing relatedness with the structural conditions involved critical reflection and awareness of the effect of historical and contemporary government policies and programs on their services. It also required negotiation for support from Aboriginal Elders and leaders, and resources, particularly funding, to transfer and deliver the program in ways that were responsive to community preferences. Community-based organizations negotiated with the provider hubs for program delivery and committed incrementally based on their ability to gain resources, particularly funding. However, a non-Aboriginal program manager from a community-based organization commented: 'there’s always logistics in our organizations to have the flexibility to do what people in communities want.’

Program evaluations documented evidence of change and were used to support funding applications, as well as adaptations of the program and related protocols and measurement tools. Thus, program evaluation resulted in what an Aboriginal facilitator and researcher, called credibility 'through a white system as well.’ The evidence for Aboriginal empowerment was translated to strengthen organizational capacity and influence local policies. A non-Aboriginal senior policy officer reflected:

'For me [FWB] has been an extraordinarily useful part of thinking about health services for disempowered people more broadly. And also some of the workforce issues, it’s been highly influential in my thinking about where we should go.’

Another Aboriginal facilitator commented: '… it just really opened my eyes—it’s bigger than a program. This is also about influencing change around policy.’

For individual FWB agents, embracing relatedness with the structural conditions also required grappling to make sense of their personal histories and to enact personal change processes. For example, an Aboriginal FWB facilitator reflected:

'You’re a product of past history, of what happens. I guess when I stand up as an Aboriginal person and go through my life’s experiences and my childhood and teenage years, and more times out of none, every Aboriginal student in that class is going to comprehend what I’m saying because they’ve had the same journey.’

Despite the success of program transfer efforts through iterative enactment of the three dimensions of embracing relatedness to support inside-out empowerment, program delivery in the majority of sites was not sustained beyond a pilot phase. A non-Aboriginal researcher and advocate reflected:

'… to try and get that level of sustainability is quite difficult for a program of the sort that we’re talking about, very difficult really. If you look across public sector programs… which are non-mainstream, to survive 10 years is quite a challenge, when you’re looking at… at least three governments in a period of time like that.’

Research respondents indicated that despite the frustrations of short term funding and their inability to sustain program delivery in many settings, program delivery had resulted in sustained benefits for individual participants. Additionally, the program’s empowerment principles had been incorporated within organizational systems and services to add value to organizational and community processes.

### Limitations

The theoretical model was based on the transfer of only one program. The inherent characteristics of the FWB program and the crucial importance of the Aboriginal Australian political and cultural conditions may make it difficult to generalize the findings of this study. However, the theoretical model was considered trustworthy. A diversity of people (involved in FWB transfer across time, place, organizational type, role, gender, and age) was interviewed; systematic comparisons were made between data and between categories; strong logical links were made between the gathered data and the argument and analysis; and those active in FWB program transfer provided feedback that the analytical interpretations made sense and offered them deeper insights about program transfer.

## Discussion

The model of program transfer and implementation, grounded in the naturally occurring process of FWB, is consistent with aspects of international implementation models. As documented in the international literature, program transfer and implementation involved individuals, organizations and interpersonal and inter-organizational networks, partnerships, and collaborations [3,4,37]. Informal networks were important for disseminating awareness of the program and cross-sectoral collaborations brought together expertise, knowledge, and resources that enabled new understandings of problems and useful program effects [4].

However, the findings of this study suggest that the implementation of innovations targeting minority Indigenous populations within developed countries may require attention to the empowering nature of the innovation and its implementation, and consideration of the multiple levels of relatedness—with self, others, and the structural conditions. First, the impetus for program transfer of supporting inside-out empowerment suggests that the relative disempowerment of Indigenous populations makes empowerment a particularly relevant attribute of the nature of interventions and the processes of their implementation. In the case of FWB, the empowering nature of the program and its implementation process made it infectious, influencing the impetus for and process of program transfer. FWB provided a framework or set of principles for engaging and empowering work with Aboriginal people at individual, group, organizational, community, and/or policy levels. These empowerment principles and attributes contributed to experiential change processes that drove participants to advocate for transfer to other settings in order to support other people’s empowerment, fostering a sense of control and ownership over the implementation process. Benefits included the development of personal empowerment, agency, capacity, a ripple effect to others, engagement in organizational and community change processes, program transfer, and value-adding to organizational, service- and policy-related endeavors at local levels. This finding suggests a need for recognition of empowerment as an impetus for program transfer and implementation and the embedding of empowering processes as an active component within processes of implementation.

Second, program transfer occurred through an organic, informal process of embracing relatedness within and between individuals and organizations, and with the broader structural conditions. FWB agents used their enhanced sense of agency from program participation and their interpersonal (family) and inter-organizational networks to influence the transfer and implementation process. The Aboriginality of providers and participants was perceived as a critical success factor for the relevance and credibility of the program. The provider hubs interacted with implementing organizations and funders to respond to demand for the program. The Western and Aboriginal structural conditions both enabled and constrained transfer, but regardless, program transfer required an active process of engagement with these broader conditions. Thus, the processes of relatedness resulted in individual agency, strengthened organizational capacity and further program transfer. The finding, however, that the program was not sustained in the majority of sites to which it was transferred suggests that recognition and sustained resourcing for coordination and linkage roles of the provider hubs could strengthen capacity for sustained program spread.

## Conclusion

Although the indicators of Aboriginal disadvantage remain daunting, program transfer and implementation offer one pragmatic and potentially cost-effective approach for incrementally improving a range of Aboriginal Australian health, wellbeing, and educational outcomes. The model of supporting inside-out empowerment by embracing relatedness suggests that factors important in the transfer and implementation of interventions to disadvantaged populations such as Aboriginal Australians, are the empowering nature of the intervention and its implementation, and the relatedness of individuals and organizations through networks and with the broader structural conditions. The theoretical model provides a new conceptual rendering of multi-levelled-relatedness as a process for transferring programs across Aboriginal Australian sites and situations. The key elements of the theoretical model provide a useful blue print to inform the transfer of empowerment-based programs on a case-by-case basis, and may be relevant beyond Indigenous populations to other disadvantaged minority populations.

## Endnote

^a^Technical and Further Education, South Australia (TAFESA) in Adelaide and Berri; Batchelor College in Alice Springs, Northern Territory; and James Cook University in Cairns and Townsville, Queensland.

## Competing interests

The author declares that she has no competing interests.

## Authors’ contributions

The author conceptualized and designed the study as a doctoral research study, and acquired, analyzed, and interpreted the data. She wrote the manuscript and was responsible for its intellectual content.
